# Induction of humoral immune response to multiple recombinant *Rhipicephalus appendiculatus* antigens and their effect on tick feeding success and pathogen transmission

**DOI:** 10.1186/s13071-016-1774-0

**Published:** 2016-09-02

**Authors:** Cassandra L. Olds, Stephen Mwaura, David O. Odongo, Glen A. Scoles, Richard Bishop, Claudia Daubenberger

**Affiliations:** 1 International Livestock Research Institute, Box 30709, Nairobi, 00100 Kenya; 2 Swiss Tropical and Public Health Institute, Socinstrasse 57, 4002 Basel, Switzerland; 3 University of Basel, Petersplatz 1, CH-4003 Basel, Switzerland; 4 School of Biological Sciences, University of Nairobi, P.O Box 30197, G.P.O, Nairobi, Kenya; 5 USDA Agricultural Research Service, Animal Disease Research Unit, Pullman, WA 99164-6630 USA

**Keywords:** *Theileria parva*, ECF, East Coast fever, *Rhipicephalus appendiculatus*, p67, TRP64, Subolesin, Histamine binding protein, Cattle

## Abstract

**Background:**

*Rhipicephalus appendiculatus* is the primary vector of *Theileria parva*, the etiological agent of East Coast fever (ECF), a devastating disease of cattle in sub-Saharan Africa. We hypothesized that a vaccine targeting tick proteins that are involved in attachment and feeding might affect feeding success and possibly reduce tick-borne transmission of *T. parva*. Here we report the evaluation of a multivalent vaccine cocktail of tick antigens for their ability to reduce *R. appendiculatus* feeding success and possibly reduce tick-transmission of *T. parva* in a natural host-tick-parasite challenge model.

**Methods:**

Cattle were inoculated with a multivalent antigen cocktail containing recombinant tick protective antigen subolesin as well as two additional *R. appendiculatus* saliva antigens: the cement protein TRP64, and three different histamine binding proteins. The cocktail also contained the *T. parva* sporozoite antigen p67C. The effect of vaccination on the feeding success of nymphal and adult *R. appendiculatus* ticks was evaluated together with the effect on transmission of *T. parva* using a tick challenge model.

**Results:**

To our knowledge, this is the first evaluation of the anti-tick effects of these antigens in the natural host-tick-parasite combination. In spite of evidence of strong immune responses to all of the antigens in the cocktail, vaccination with this combination of tick and parasite antigens did not appear to effect tick feeding success or reduce transmission of *T. parva*.

**Conclusion:**

The results of this study highlight the importance of early evaluation of anti-tick vaccine candidates in biologically relevant challenge systems using the natural tick-host-parasite combination.

**Electronic supplementary material:**

The online version of this article (doi:10.1186/s13071-016-1774-0) contains supplementary material, which is available to authorized users.

## Background

During tick feeding saliva proteins are secreted into the feeding lesion in the skin of the vertebrate host. The saliva of ticks has been shown to contain an array of biologically active proteins with functions essential to feeding success [1]. Among other things these include: (i) immunosuppressive components to prevent the host from rejecting the tick and to suppress inflammation and irritation thus reducing the chances of being groomed off; (ii) anticoagulants and vasodilators to prevent blood clotting and keep blood flowing; (iii) cement cone proteins to ensure attachment to the host [2]. Repeated host exposure to tick feeding has been shown to result in the development of resistance against future infestations, most likely through development of an adaptive immune response targeting the saliva proteins [1–5]. This natural ability to develop resistance to ticks forms the conceptual basis for development of vaccines targeting tick feeding. Vaccinating with tick saliva antigens can induce production of antibodies that bind to and interfere with the function of the proteins in tick saliva. If particular saliva proteins are essential and have non-redundant functions, antibody binding should abrogate their functions and reduce feeding success. If antibodies to the particular saliva protein can block the function of essential saliva proteins they can make the feeding site a hostile environment for the tick and for the pathogens in such a way that effective feeding and transmission cannot take place. In addition, interference with saliva components that inhibit itch and inflammation and aid attachment may make the feeding ticks more subject to being groomed off. Either of these mechanisms may result in tick mortality, reduction in tick feeding success and/or reduced reproductive capacity, which may in turn reduce transmission of tick-borne disease [6–9].

Although a number of specific tick antigens have been proposed as potential transmission blocking vaccine candidates, no commercially available anti-tick feeding vaccine has been developed. Instead, tick vaccine studies have focused on concealed targets in the tick gut which induce mortality by damaging the gut and interfering with blood meal digestion. Bm86 is a tick gut protein that has been used as a commercial vaccine. These vaccines have been commercially available (Tick-Guard, Gavac), but their efficacy has been spotty and unpredictable and they have seen limited use [10, 11]. The value of anti-tick vaccine candidates like Bm86 can be established by examining tick feeding success and survival but it has been particularly difficult to establish the value of these antigens in comparison to other candidates because there is no standardized protocol for evaluation of anti-tick vaccine efficacy. Apart from the obvious differences in antigen preparation, formulation and administration there are also differences in tick and host species and type of challenge used in vaccine trials. Antigens which prove effective in non-natural host models may behave very differently when evaluated using natural tick-host combinations. Vector-borne host-tick-pathogen relationships co-evolved over long periods of time and antigens proving effective against one species of tick may not prove effective against another.

East Coast fever (ECF) caused by the protozoan parasite *Theileria parva* is considered the most devastating tick-borne disease of cattle in sub-Saharan Africa [12–14]. *Theileria parva* is transmitted by the three-host tick *Rhipicephalus appendiculatus*. Infection with this parasite results in high rates of mortality and morbidity [12–14]. The development of a sustainable control method for ECF is critical for increased livestock production in affected areas [13]. Using the ECF as a model system, we attempted to interrupt successful tick feeding by targeting different aspects of tick physiology through vaccination with tick antigens. The antigens were chosen based on previous published work that either demonstrated a measurable anti-tick effect in other host/tick species or showed potential as an anti-tick antigen based on a demonstrated affect that may interfere with tick feeding. Antigens chosen included two female and one male-derived *R. appendiculatus* histamine binding protein, the cement cone protein TRP64, subolesin and p67C. Tick histamine binding protein is thought to sequester host histamine in the feeding lesion, neutralizing host inflammation and immune responses, reducing the likelihood of rejection from the feeding site and manual removal by grooming [15]. TRP64 was identified as a *R. appendiculatus* cement cone protein, antibodies to which bind to both epitopes present in the saliva as well as within the midgut [16]. The cement cone is essential to anchor the tick to the host skin [1, 2]. Subolesin has been identified as an intracellular regulatory protein with a role in signal transduction and vaccination against this protein has shown anti-tick effects on other tick species [11]. In addition to tick antigens, *T. parva* sporozoite surface antigen p67C, has shown variable efficacy in previous studies and it was included here to see if it could act in a cumulative manner with the other antigens to reduce transmission efficiency by interfering with parasite entry into bovine host cells [17–19]. Although each of these proteins alone may have an effect on feeding success and transmission, we hypothesized that the combination of several different antigens that may interfere with saliva function and reduce transmission efficiency through an incremental effect. For each of the candidate anti-tick components, this is the first time they have been tested in the natural host-tick-parasite model.

## Methods

### Vaccine antigen expression and purification

**Fig. 1:**
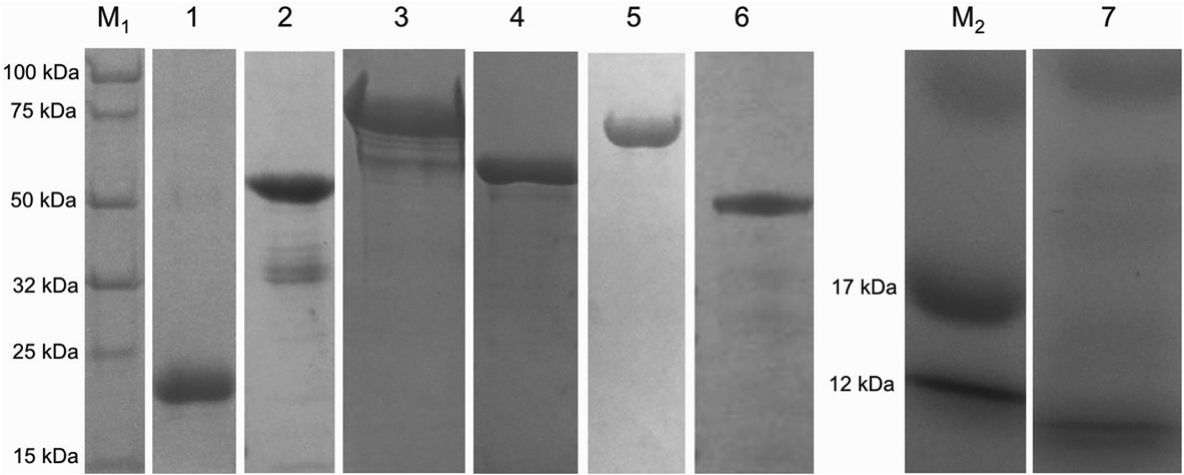
Coomassie blue stained gel showing SDS-PAGE analysis of the purified recombinant antigens incorporated into the multivalent vaccine. M_1_, Protein Ladder; Lane 1, Histamine binding protein (male variant); Lane 2, Histamine binding protein (female variant 1); Lane 3, Histamine binding protein (female variant 2); Lane 4, TRP64 (truncated); Lane 5, TRP64 full length; Lane 6, Subolesin; Lane M_2_, Protein Ladder; Lane 7, p67C

Antigens selected for the multivalent cocktail included three *R. appendiculatus* histamine binding proteins [male (HBPM, AAC63108.1), female-one (HBPF1, AAC63106.1) and female-two variants (HBPF2, AAC63107.1)] [15], two different *R. appendiculatus* cement cone protein antigens [TRP64 [full length (TRPFL, AF469170.1) and TRP truncated variants (TRP18-89, amino acids 18-89)] [16], the *R. appendiculatus s*ubolesin homologue (4D8, ABA62331.1) and *T. parva* sporozoite antigen p67C [19]. Using the above accession numbers, nucleotide sequences for each antigen were retrieved from GenBank (http://www.ncbi.nlm.nih.gov/genbank/) and submitted to GenScript Corp. (New Jersey, USA) for expression and purification. Antigens were expressed in *E. coli* with either 6× His or TF tags and purified by affinity chromatography. Subolesin was expressed with a GST tag, which was removed prior to vaccination of cattle. The p67 C-terminal (p67C) antigen was cloned in pQE30 and expressed in *E. coli* as outlined in Bishop et al. [19]. Due to the small size of p67C, 80 amino acids (≈10 kDa), size exclusion chromatography was used for purification. Antigens were quantified using the Bradford protein assay with BSA as a standard. All antigens were determined to have a purity of 75 % or greater by SDS PAGE and Coomassie blue staining (Fig. 1).

### Vaccination of cattle with the multivalent cocktail

Thirty *Bos tarus* (either Friesian or Friesian/Ayeshire cross) calves, 3 months of age, were randomized into two groups, vaccinated (*n* = 20) and control (*n* = 10). Calves were raised and maintained under strict tick-free condition and serologically tested prior to use to confirm they were free of tick-borne infections (babesiosis, theileriosis and anaplasmosis). The seven antigens were separated into two different pools of inocula for vaccination to reduce the potential for antigenic competition between vaccine components. The first pool contained subolesin, HBPF2, TRP18-89 and p67C and was administered subcutaneously over the left shoulder. The second pool contained HBPM, HBPF1, TRPFL and was administered subcutaneously over the right shoulder. Inocula contained 50 μg of each antigen with the final volume adjusted to 1 ml with phosphate buffered saline (PBS) pH 7.4. Preparations were emulsified in an equivalent 1 ml volume of Montanide ISA 50 V adjuvant (Seppic, Paris, France) mixed according to the manufacturer’s instructions. Each animal received three inoculations separated by four-week intervals (days 0, 28 and 56) for a total of 150 μg of each antigen. Control preparations consisted of 1 ml of PBS emulsified in 1 ml of Montanide ISA 50 V adjuvant. Control preparations were administered subcutaneously over the left and right pre-scapular region in the same way as the vaccination group. Serum samples were collected from the jugular vein before the first vaccination and at two-week intervals thereafter until the end of the study. Serum was stored at -20 °C until tested by ELISA for reactivity against each individual antigen.

### Monitoring humoral immune response to vaccination by indirect ELISA

Humoral immune responses directed against each antigen were monitored for each animal. Ninety six-well plates (Polysorp, Nunc) were coated with 0.5 μg recombinant protein per well and incubated for one hour at 37 °C. All washing steps were carried out using Phosphate Buffered Saline (10 mM PO_4_^3−^, 137 mMNaCl and 2.7 mM KCl) containing 0.5 % (v/v) Tween 20 (PBST). Plates were washed three times in an automated plate washer for each wash step. Plates were blocked with 1 % casein in PBST for 30 min at 37 °C. Serum was applied at an initial concentration of 1:500 followed by 1:2 dilutions and incubated for one hour at 37 °C. After washing, anti-bovine IgG, whole molecule, peroxidase conjugate (Sigma, St. Louis, USA) was added at a dilution of 1:10,000 in PBST and incubated for one hour at 37 °C. Plates were washed and given a final rinse in PBS. Plates were developed using SIGMAFAST OPD (Sigma, St. Louis, USA) following instructions and signals were evaluated at optical density (OD) 450 nm in a plate reader. Mean readings (with standard error) were calculated for each time point by grouping control and vaccinated animals. Endpoint titres were determined as the last serum dilution where the OD of test sera was ≥ 2 OD of negative control bovine serum donor. Time points evaluated were before vaccination and two weeks after each inoculation (days 0, 14, 42 and 70).

### Evaluation of the effect of vaccination on tick feeding and transmission of *T. parva*

Two different tick strains were used in this study. The *R. appendiculatus* Muguga laboratory tick line [20] was used to assess the effect of vaccination on tick feeding success. The Muguga ‘low-line’ tick colony was used to transmit *T. parva* parasites to cattle to assess the effect of vaccination on transmission. The ‘low-line’ colony was originally developed and has been maintained at the International Livestock Research Institute (ILRI) Tick Unit since 1994; this colony was selected from the Muguga laboratory tick line by cross-breeding siblings with reduced susceptibility to *T. parva* infection [21]. Normal uninfected Muguga colony ticks were routinely reared on rabbits and cattle, and maintained in Biological Oxygen Demand (BOD) incubators at 28 ± 1 °C when not feeding on hosts. Infected ‘low-line’ ticks for the transmission component of the study were produced as described in Odongo et al. [22]; briefly, ticks were infected with *T. parva* by feeding as nymphs on an infected calf and after molting to adults were maintained until used for transmission in BOD incubators at 24 ± 1 °C, 80 % relative humidity.

### Evaluation of the effect of anti-tick vaccination on tick feeding and tick reproductive capacity

Two weeks following the final vaccination, normal colony ticks were applied to each calf in two separate tick-feeding bags attached with adhesive to the skin of each calf. One bag was attached to the back of the calf and contained 200 nymphs. Anti-tick effects on nymphs were measured as engorgement weight (average for 100 ticks) and the proportion of nymphs successfully molting to adult. A second feeding bag was attached to the base of the left ear and contained 50 adult females and 50 adult males. Assessment of the effect of vaccination on female feeding success included: mortality rate (number replete out of 50 applied); average engorgement weight of each female and egg laying/hatching capacity. Male ticks were only applied to stimulate female feeding and were not used as part of the anti-tick assessment.

### Evaluation of the effect of vaccination on transmission of *T. parva*

The vaccine cocktail was evaluated by exposing cattle to *T. parva* infected ‘low-line’ ticks. The *T. parva* infection rate in the female tick population used for challenge was calculated to be 20 % with the average of 5.8 infected acini per infected tick based on stained salivary gland smears [23]. Thirty *T. parva* infected female ‘low-line’ ticks were placed together with thirty uninfected male ticks in an ear bag attached to the base of the right ear of each calf. Uninfected male ticks were added only to stimulate the feeding of female ticks and did not contribute to parasite burden. Female ticks were allowed to feed till repletion after which engorgement weight and egg laying capacity were measured. Transmission was measured as the ability of adult females to feed successfully and transmit *T. parva* to calves. Rectal temperature was recorded daily after tick application, pyrexia was defined as a rectal temperature above 39.5 °C. Transmission of *T. parva* parasites and the establishment of infection was evaluated using a combination of: (i) microscopy; (ii) PCR and (iii) serology. Transmission was deemed to have occurred if antibodies to *T. parva* could be detected along with PCR detection and/or microscopic identification of parasites. In the event of acute disease calves were treated as required with short- or long-acting oxytetracycline (Copermycin or Butalex®, respectively).

#### Microscopy

Lymph node biopsies were taken daily from day ten after tick challenge in the local lymph node draining the site of infection (right parotid lymph node), and from day 15 in the contralateral left pre-scapular lymph node. Perioheral blood was collected from the ear vein. Lymph node aspiration smears and blood smears were stained with Geimsa and examined for the detection of schizonts and piroplasms respectively [24].

#### PCR

Blood samples for PCR analysis taken from each calf two weeks after infection. DNA was extracted using the DNeasy Blood and Tissue kit (Qiagen, Hilden, Germany) according to instructions. The p104 gene PCR parameters and the primers used in the primary PCR were as described previously [25, 26]. All reactions were performed in 15 μl volumes using 5 μl of DNA extracted from blood for the primary reaction or 5 μl of the primary PCR reaction for the nested PCR reaction with 0.25 units of Taq DNA polymerase, (Promega, Madison, USA), 1× PCR buffer (Promega), 200 mM of each dNTP (Promega) and 25 ng of primers and 1.5 mM of MgCl_2_. The final PCR products were visualized by Ethidium bromide staining and UV trans-illumination.

#### Serology

Antibodies against the *T. parva* PIM antigen were detected using the PIM-ELISA developed at ILRI, as previously described [27]. Serum samples used for detection were taken 12 weeks after tick application (in cases where animals died or were euthanized, the last sample before death was used for serology).

## Results

Two animals were removed from the analysis of the transmission experiment due to concurrent lungworm infections which could potentially exacerbated clinical signs of East Coast fever. However, ticks collected from these animals were still included in the evaluation of anti-tick effects.

### Antibody titres

**Fig. 2:**
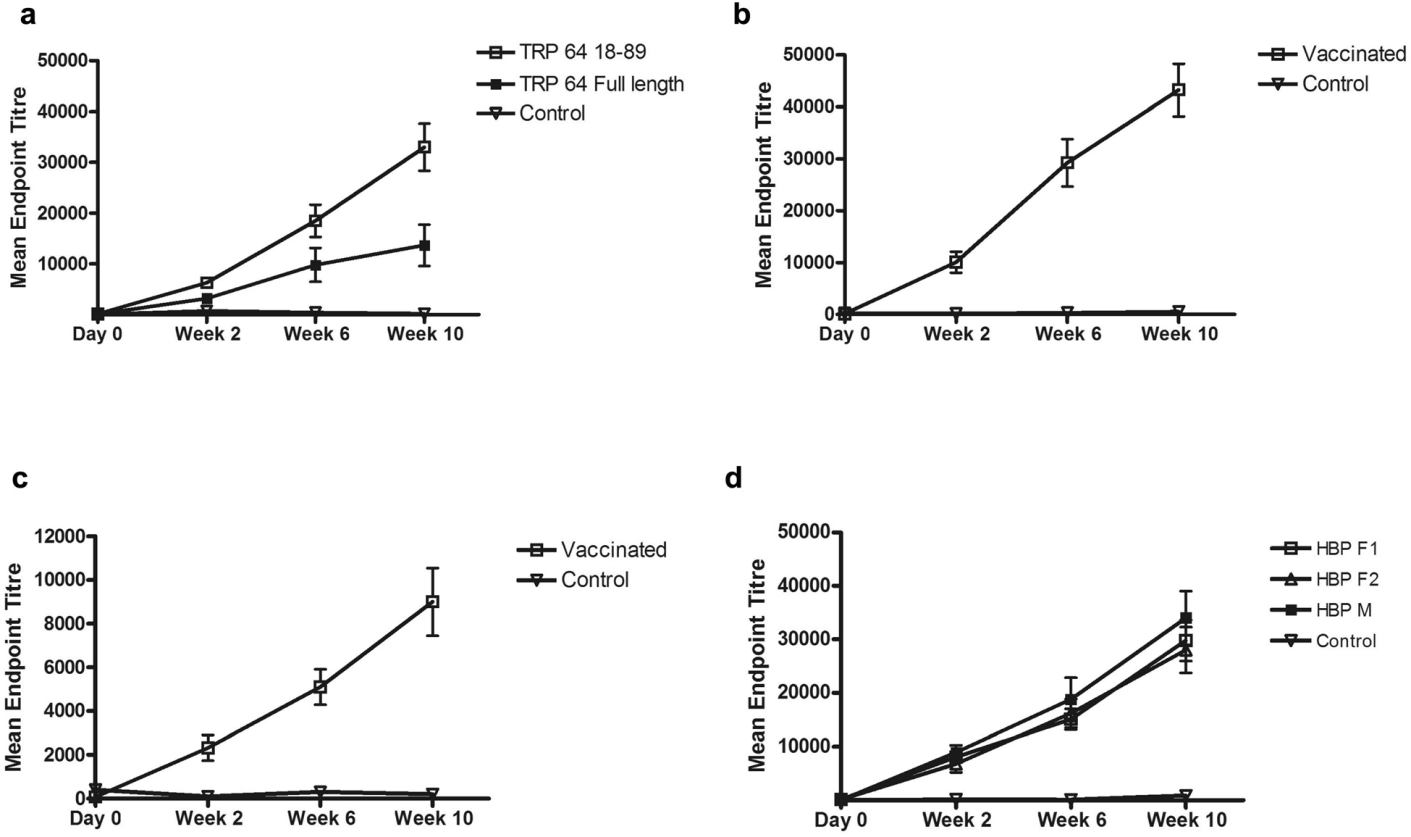
Development of antibody titres against vaccine antigens pre- and post-vaccination. Time points evaluated included pre-vaccination (day 0), two weeks after first inoculation (week 2), two weeks after second inoculation (week 6) and two weeks after third inoculation (week 10). **a** TRP64. **b** Subolesin. **c** p67C. **d** Histamine binding protein

Antibody titres against each antigen could be detected in all vaccinated animals (Fig. 2; Additional file 1: Table S1). There was a large difference between the antigenicity of the truncated and full-length versions of TRP. The average titre for the full length version of TRP64 was 1:10,700 compared to 1:33,000 for the truncated TRP. There were roughly equal responses to the histamine binding protein variants, between 1:30,000 and 1:38,800, with marginally higher titres developed to the male variant. High antibody titres were generated against subolesin (average endpoint titres of 1:40,600) but lower titres were observed against p67C with average endpoint titres of only 1:8,500.

### Anti-tick effect of vaccination

Despite demonstrable antibody titres to each component of the anti-tick vaccine cocktail, no significant anti-tick effects were observed in the normal Muguga colony ticks (Table 1, Additional file 1: Table S2). Nymphal ticks fed equally well in both groups with the average weight of 100 pooled nymphs being 1.2 g for cattle in both the vaccinated group (*n* = 20) and control group (*n* = 10). Nymph molting was not inhibited in the vaccine group with 98.1 % successfully compared to 97.9 % in the control group (Mann-Whitney *U* = 100; n_1_ = 20, n_2_ = 10; *P* = 0.351; two-tailed). The multivalent cocktail had no effect on adult female survival with an average of 47 of 50 ticks from the vaccinated group and 46 of 50 collected in the control group (Mann-Whitney *U* = 79; n_1_ = 20, n_2_ = 10; *P* = 0.221; two-tailed). Female ticks from the vaccinated group laid on average 304 mg of eggs compared to ticks in the control group females which laid 246 mg each (Mann-Whitney *U* = 56.5; n_1_ = 20, n_2_ = 10; *P* = 0.056; two-tailed) There was little variation in tick feeding success within the vaccinated group ticks and no correlation between tick biological parameters and antibody titre to specific antigens could be established.

**Table 1:** Evaluation of the effect of multivalent anti-tick vaccine on the biological fitness of normal *Rhipicephalus appendiculatus* adult ticks and nymphs from the Muguga colony

	Vaccinated cattle hosts (*n* = 20)^a^	Control cattle hosts (*n* = 10)^a^	*U*-value^b^	*P*-value^b^
Nymphs				
Average weight of 100 nymphs	1.2 ± 0.0	1.2 ± 0.0	100.0	< 0.999
Nymphs molting successfully of 100^c^	98.1 ± 0.4	97.9 ± 0.4	79.0	0.351
Adults				
Number of engorged females recovered from 50 applied	46.5 ± 1.3	45.9 ± 1.0	71.5	0.208
Average adult female replete weight (mg)	528.0 ± 12.0	548.0 ± 9.0	69.5	0.187
Total egg weight laid (g)	13.4 ± 0.6	11.5 ± 0.9	64.0	0.117
Average egg weight/tick (mg)	304 ± 17	246 ± 15	56.5	0.056

^a^Values represent the mean ± standard error of the mean (SEM)

^b^Mann-Whitney test: vaccinated *vs* control group

^c^Percentage of nymph ticks successfully molting to the adult stage in a random population of 100 individuals collected for monitoring

The effect of vaccination was more pronounced in the infected Muguga ‘low-line’ ticks. Ticks fed less effectively on vaccinated hosts in almost all parameters measured (Table 2; Additional file 1: Table S3). Ticks collected from vaccinated cattle laid 67 ± 40 mg of eggs per tick compared to control ticks (83 ± 34 mg) (Mann-Whitney *U* = 75.5; n_1_ = 20, n_2_ = 10, *P* = 0.383; two-tailed). An average of 15 replete females were recovered from vaccinated cattle (14.9 ± 7.6) compared to 18 from control cattle (17.8 ± 7.6) (Mann-Whitney *U* = 81.5; n_1_ = 20, n_2_ = 10; *P* = 0.550; two-tailed). Female ticks collected from vaccinated cattle weighed 238 ± 79.0 mg compared to ticks collected from control animals (275 ± 30.0 mg) (Mann-Whitney *U* = 78.5, n_1_ = 20, n_2_ = 10; *P* = 0.462; two-tailed). Overall, ‘low-line’ ticks may have been more sensitive to vaccine-induced antibodies than normal colony ticks. In general, the ‘low-line’ ticks did not feed as well as normal colony ticks and for the tick parameters measured, there was more variation between individual animals for ‘low-line’ ticks than normal colony ticks. This could not be correlated with antibody titre and most likely due to other factors such as *T. parva* infection level or ‘low-line’ strain biology [22].

**Table 2:** Comparison on the feeding efficacy of *Theileria parva*-infected *Rhipicephalus appendiculatus* Muguga ‘low-line’ ticks

	Vaccinated^a^	Control^a^	*U*-value^b^	*P*-value^b^
Number of engorged females recovered from 30 applied	14.9 ± 7.6	17.8 ± 7.6	81.5	0.550
Average adult female replete weight (mg)	238 ± 79.0	275 ± 30.0	78.5	0.462
Total egg weight laid (g)	1 ± 0.6	1.4 ± 0.7	59.5	0.106
Average egg weight/tick (mg)	67 ± 40	83 ± 34	75.5	0.383

^a^Values represent the mean ± standard error of the mean (SEM)

^b^Mann-Whitney test: vaccinated *vs* control group

### Evaluation of the effect of vaccination on transmission of *T. parva*

The *T. parva* infection rate in the female tick population used for challenge was calculated to be 20 % with the average of 5.8 infected acini per infected tick. Based on an infection rate of 20 % in the tick population we would estimate that each animal was challenged by at least ≈ 6 ticks with infections discernable by smear analysis. Detection of *T. parva* in cattle after exposure to infected ticks was measured using three methods: microscopy, serology and PCR. Transmission was deemed to have occurred if antibodies to *T. parva* antigen PIM could be detected in addition to either (or both) PCR detection of parasite DNA or microscopic presence of parasites in blood/lymph node smears. Transmission of *T. parva* from infected ticks was confirmed in each animal indicating that vaccine-induced transmission blocking did not occur (Table 3).

**Table 3:** Development of East Coast fever symptoms in cattle vaccinated with the multivalent antigen cocktail after exposure to *Theileria parva*-infected ‘low-line’ ticks

		Vaccinated cattle (*n* = 18)	Control cattle (*n* = 10)	*P*-value
Pyrexia^a,b^	Number of animals where symptom observed (%)	15 (83 %)	9 (90 %)	< 0.999^d^
	Day of first onset	10.3 (1.2)	11.1 (1.8)	0.801^e^
	Duration	5.8 (0.9)	5. 6 (1.4)	0.803^e^
Regional lymph node parasitosis^a^	Number of animals where symptom observed (%)	13 (72 %)	9 (90 %)	0.375^d^
	Day of first onset	13.5 (0.6)	14.0 (1.1)	0.774^e^
	Duration	4.2 (0.7)	3.7 (0.9)	0.547^e^
Contra-lateral lymph node parasitosis^a^	Number of animals developing symptom (%)	8 (44 %)	6 (60 %)	0.695^d^
	Day of first onset	16.1 (0.5)	17.2 (0.6)	0.211^e^
	Duration	3 (0.8)	2.5 (1.7)	0.825^e^
Piroplasm^a^	Number of animals where symptom observed (%)	5 (28 %)	4 (40 %)	0.678^d^
	Day of first onset	17.6 (0.6)	17.0 (0.4)	0.722^e^
	Duration	3 (0.6)	3 (0.7)	< 0.999^e^
Nested p104 PCR detection of parasites		17 (94 %)	9 (90 %)	< 0.999^d^
PIM antibodies	Number of animals where antibodies detected (%)	18 (100 %)	10 (100 %)	< 0.999^d^
	Average PP value^c^	44.7 ± 16.1	53.4 ± 22.8	0.249^e^

^a^Values are displayed as the average day of first detection (SEM). Where symptoms were not observed in an animal, no values are reflected

^b^Pyrexia was defined as rectal temperature exceeding 39.5 °C

^c^The average Percentage Positive (PP) value calculated as the (OD of test sample/OD of strong positive) × 100

^d^Fischer’s exact test

^e^Mann-Whitney test

**Fig. 3:**
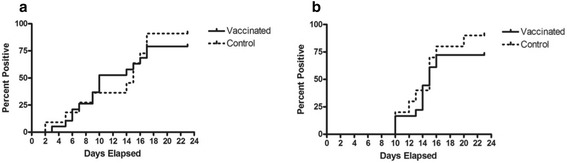
Kaplan-Meier curves for time of first onset of ECF clinical signs in vaccinated and control cattle, **a** showing development of pyrexia (temperature above 39.5 °C) and **b** showing the identification of schizont stage parasites in the lymph node draining the site of infection

Kaplan Meier plots (Fig. 3) show that while transmission blocking did not occur; slightly milder disease clinical signs were observed in the vaccinated group although this difference was not statistically significant. No clinical signs of ECF disease were detected in two animals in the vaccinated group (BF003 and BF030, Additional file 1: Table S4). Both cattle showed no visible parasitemia (either shizonts or piroplasms) or pyrexia response while all animals in the control group showed at least one clinical sign of infection. Although no correlation between anti-p67 titre and severity of disease was observed, calf BF003 and BF030 did have anti p67 endpoint titres of 1:12,000 and 1:16,000 respectively, both above the group average of 1:8,500. Fewer animals in the vaccinated group 83 % (15 out of 18) compared to 90 % (9 out of 10) control animals developed pyrexia (Table 3). During East Coast fever infections, parasites infecting lymphocytes (schizonts) are disseminated through the body of the infected animal. Presence of schizont stage parasites in the lymph node draining the site of infection indicates establishment of infection. Schizonts also were detected in 90 % (9 out of 10) control animals and in 72 % (13 of 18) vaccinated animals (Table 3).

## Discussion

It was hypothesized that the anti-tick feeding and anti-parasite effects of the components of this multivalent cocktail would act cumulatively, leading to a reduction in tick feeding and thereby *T. parva* transmission. Tick saliva components contained in the vaccine aimed to reduce or inhibit tick feeding. With feeding reduced, exposure of the host to tick-borne pathogens would in turn also be reduced. Through the action of the parasite component, anti-parasite antibodies would target and serologically neutralize any parasites entering the bovine host inhibiting infection of lymphocytes thereby block parasite transmission to the host [17–19]. The anti-tick effect of the vaccine cocktail was evaluated in both nymph and adult female *R. appendiculatus* ticks. *Theileria parva* infection rates are highest in adult females they are considered the most important stage for acute disease transmission. Nymphal ticks have lower infection levels and are considered important for the transmission of sub clinical disease and as a parasite acquisition stage for adult ticks [28]. The effect of the vaccine focused on the ability of nymph and adult female ticks to successfully feed and for adult females to transmit *T. parva*.

As no significant anti-tick effect was observed it can be assumed that vaccination had no impact on tick feeding and host exposure to the parasite was not reduced through vaccination. All tick antigens selected for this study had a published history of being effective anti-tick vaccine candidates or showed a potential role in successful feeding in other tick-host models. Tick subolesin was first identified in *Ixodes scapularus* and its efficacy as an anti-tick vaccine has been evaluated in a number of tick species using a variety of approaches. In *I. scapularis*, subolesin has a proposed role as an intracellular regulatory protein involved in signal transduction [11]. Vaccination with subolesin has been shown to protect against *I. scapularis* infestations, resulting in reduced tick survival, feeding and reproduction [29–43]. Interference with subolesin through either vaccination or RNAi reduced tick infection rates with pathogens *Anaplasma marginale*, *Anaplasma phagocytophilum* and *Babesia bigemina* [36–38]. The effect of vaccination with subolesin on *R. appendiculatus* has not been investigated but showed promising results for the control of cattle tick species *R. microplus* and *R. annulatus* [37–39]. Both subolesin-sensitive species *R. microplus* and *R. annulatus* are one-host tick species, feeding on a single host from larvae till engorged adults. This is in contrast to *R. appendiculatus*, a three-host tick which feeds on a separate host at each stage. This stark difference in feeding behavior provides one-host tick species a much longer exposure period for antibodies targeting tick antigens to bind to and cause damage compared to a three-host tick species. Even where subolesin has been shown effective as a tick control antigen, tick species-specific vaccine effects vary. The anti-tick effect of subolesin vaccination on *R. microplus* was predominantly reduction of tick infestation with a small reduction in egg fertility [35, 42, 43]. In contrast, *R. annulatus* primarily showed reduced oviposition and egg fertility with lower reductions in infestations [42]. From the current study we have concluded that vaccination with subolesin is unlikely to be useful as a control method for *R. appendiculatus*.

TRP64 (64TRP) was identified as a *R. appendiculatus* cement cone protein, antibodies to which bind to both epitopes present in the saliva as well as within the midgut [16]. Sera raised against various truncated versions of TRP64 cross reacted with tissue extracts from *Rhipicephalus sanguineus*, *Ixodes ricinus*, *Amblyomma variegatum* and *R. microplus* [44]. Anti-tick effects against non-target tick species *R. sanguineus*, *I. ricinus*, *A. variegatum* and *R. microplus* were observed after 64TRP vaccination of guinea pigs [44, 45]. *Rhipicephalus appendicualus* ticks fed on TRP64 vaccinated guinea pigs showed increased mortality, decreased mean engorgement weight and decreased egg-laying mass [16]. Vaccination of rabbits with TRP64 did not produce a measurable anti-tick effect on *R. appendiculatus* [46]. Vaccination of mice with 64TRP was shown to inhibit tick feeding as well as reduce levels of transmission of tick-borne encephalitis virus by *I. ricinus* making it a transmission blocking vaccine candidate [47]. None of the anti-tick effects demonstrated for 64TRP in small-animal models could be replicated in our experiment using the natural host-vector system despite the induction of high antibody titres to both TRP64 variants in cattle.

Histamine binding proteins contained within the tick saliva are thought to play a role in controlling the inflammation and itch-responses by sequestering histamine at the feeding site competing with host histamine receptors [15]. Although not previously evaluated as an anti-tick vaccine candidate, *R. appendiculatus* histamine binding protein was able to prevent murine allergic asthma [48]. By inhibiting tick histamine binding protein, the feeding site may become a hostile environment leading to a reduction in tick feeding. In this study, cattle were restrained by the head in stalls during tick feeding. This restraint would have made them unable to groom, even if the itch response had been greater in the vaccinated group. The components of tick saliva are highly redundant [1] and even if a component is completely neutralized, it is possible that other molecules performing the same or similar functions are present. Although saliva antigens are attractive targets for anti-tick vaccination, this approach may be unlikely to succeed due to the complexity of tick saliva.

The lack of protection observed after vaccination may be as a result of a number of factors. The observed differences in anti-tick effects between the antigens used here and those reported in other host-vector systems may be partly related to specific tick antigen recognition patterns by the host species. Tick components detected by the experimental non-native host species may not be immunogenic in the natural host-tick system [1]. The varying epitopes recognized by different host species after vaccination may in part account for the differences in anti-tick effects. This phenomenon is well illustrated when *R. sanguineus* ticks, which naturally feed on dogs, are fed on guinea pigs. In guinea pigs, resistance to *R. sanguineus* results after a low number of tick bites, characterized by high tick mortality, reduced engorgement weights and reproductive capacity likely due to their natural resistance to ticks. In stark contrast, repeated exposure of dogs to *R. sanguineus* results in an immediate inflammatory response in the skin with a delayed hypersensitivity response and little resistance [49–51].

Although inoculation of antigens was split over two injection sites to avoid antigenic competition, it is possible that competition still occurred. Studies have shown that antigenic competition occurs when multiple antigens are simultaneously inoculated even when using separate injection sites [52]. Lower antibody titres to each antigen are developed and overall vaccine efficacy is reduced [52–54]. As five of the seven tick antigens are variants of each other (male and female histamine binding proteins; full and truncated versions of TRP64) competition likely still have occurred even with separate inoculation sites. As a result, the actual individual anti-tick effects of each of these antigens in the *R. appendiculatus*-cattle model still remains unknown. Due to the redundant nature of tick saliva, balancing redundancy with antigenic competition will be important in future saliva based anti-tick vaccine studies. In addition to antigenic competition, the choice of adjuvant and protein expression system used in this study would undoubtedly play a role in the ultimate success of these antigens as tick control candidates.

As no anti-tick effect could be demonstrated with the antigen cocktail, any transmission blocking would be due to the action of the anti-parasite component of the vaccine. The *T. parva* antigen p67 is expressed on the sporozoite surface and is involved in parasite entry into bovine lymphocytes [18]. Results obtained from experimental trials with p67 showed that vaccination could protect between 60 and 70 % of cattle from lethal sporozoite needle challenge [18, 19, 55]. Under field-based tick challenge (determined to be low to moderate in intensity), the vaccine failed to achieve previously reported levels of protection. In three geographically separate trial sites, two showed no significant reduction of severe ECF. In the third site, a significant reduction of severe ECF by 30 % was observed [56]. Needle challenge experiments run concurrently with field trials showed a decrease in the incidents of severe ECF by approximately 50 % suggesting that protective efficacy varies between natural tick challenge and artificial needle challenge [56]. Of all the antigens administered in this study, antibody titres targeting p67 C were the lowest. An increase in titres may be achieved through increasing the amount of antigen used to vaccinate cattle (previous p67 vaccination studies administered a total of 450 μg of antigen, compared to 150 μg used in this study) or changing the vaccine formulation. It is possible that antigenic competition with tick antigens also contributed to the poor response to p67. Parasites were successfully transmitted to each calf confirmed by anti-PIM antibodies, microscopy and/or PCR indicating that parasite blocking was not successfully achieved. The onset and severity of ECF disease clinical signs is related to the number of parasites inoculated into the host. Cattle receiving higher numbers of sporozoites generally develop clinical signs earlier with higher parasitemia than those receiving lower doses [12, 57]. As the exact number of sporozoites each animal received through tick feeding is unknown, it is difficult to know if animal BF003 and BF030 did not develop disease clinical signs due to their higher p67 antibody titres or a chance lower tick infection rate. BF025 had the highest anti-p67 titre (1:32,000) and still develop schizont parasitosis in both regional and contra-lateral lymph nodes suggesting that the individual tick challenge received by each animal is an important factor in disease severity in this study.

The only meaningful evaluation of this combination of tick and parasite antigens had to employ natural feeding of infected ticks to deliver infectious parasites. Although the ‘low line’ used in this study was bred to have a low susceptibility to infection this is compared to a laboratory line with high susceptibility to infection [21]. After feeding on an acutely parasetimic host, infection rates in the artificially selected ‘low-line’ are still higher than animals would likely be exposed to under field conditions. Reported field infection rates in East Africa are generally low with less than 5 % of the tick population infected and infected ticks show low levels of infection, around 1 infected acini per tick [58–61]. The tick population used in this study had a 20 % infection rate with individual tick infections ranging from 1 and 24 infected acini per tick. Furthermore, Odongo et al. [22] showed that nested PCR was able to detect more infected ticks than microscopy, suggesting that the estimated challenge dose of 6 infected ticks in this study may be a considerable underestimate of the actual exposure in this experiment. In future, the development of a tick challenge model more representative of what animals would encounter under natural field conditions is important. This should involve feeding non-selected tick lines on persistently infected cattle with lower parasitemia apposed to selected lines fed on acutely parasitemic hosts. This will allow the production of tick challenge material with a more uniform infection rate reducing the sporozoite dose variation experienced by individual cattle. Such a challenge model would make study results easier to interpret and allow protective antibody titres to be identified.

It was interesting to note that the effect of vaccination on tick feeding differed between the two tick strains used in this study. In general, ticks from the Muguga ‘low-line’ are routinely smaller than Muguga normal colony ticks and it is thought that less effective feeding contributes to the lower *T. parva* infection rates in the ‘low-line’ [22]. Subolesin vaccination resulted in reduced oviposition in *R. annulatus* [42] and it is possible that antibodies targeting subolesin accounted for the reduced egg laying capacity in the Muguga ‘low-line’ ticks. Why it does not affect the normal colony in the same manner is unknown, but the results highlight that anti-tick effects may vary between strains of the same tick species. The implication of this being that anti-tick effects on field tick populations may be vastly different to those observed when using laboratory strains.

## Conclusion

A combination tick-parasite multivalent vaccine was evaluated for its effect on *R. appendiculatus* feeding and *T. parva* transmission. This is to our knowledge the first report of the evaluation of these antigens in the biologically relevant tick-host-pathogen system. Both, the anti-tick and transmission blocking potential of this vaccine was not significant enough to merit further pursuit of this specific antigen combination in the current formulation. Although candidates were selected to act in a cumulative manner, antigenic competition between antigens may have reduced the efficacy of antigens. The effect of the individual antigens remains unknown and changes in vaccine formulation may improve the anti-tick effect. Interestingly, anti-tick vaccine effects varied between tick strains, suggesting differences between the Muguga ‘low-line’ and normal colony despite originating from the same parent stock. Together, the data from this study highlights the importance of early evaluation of any proposed anti-tick antigen in the natural host-tick system for a more accurate representation of the likely effects.

## Additional file

Additional file 1: Table S1. Endpoint ELISA titres for each animal at the time of tick application. Values represent the last serum dilution where the OD of test sera was ≥ 2 OD of negative control bovine serum donor. **Table S2.** Tick biological data measurements for normal *Rhipicephalus appendiculatus* Muguga normal colony ticks from individual animals after multivalent anti-tick vaccination. **Table S3.** Tick biological data measurements for *Rhipicephalus appendiculatus* Muguga ‘low-line’ ticks from individual animals after multivalent anti-tick vaccination. **Table S4.** Summary of the East Coast fever symptoms in individual animals after exposure to 30 ‘low line’ Muguga ticks infected with *Theileria parva*. (*DOCX 43 kb*)

## Abbreviations

ECF: East coast fever
ILRI: International Livestock Research Institute.
