# Supplementary material for: Induction of humoral immune response to multiple recombinant Rhipicephalus appendiculatus antigens and their effect on tick feeding success and pathogen transmission
Source: Parasit Vectors. 2016 Sep 2;9(1):484. doi: 10.1186/s13071-016-1774-0 (PMC5010713; doi:10.1186/s13071-016-1774-0)
Supplement: Additional file 1: Table S1. — Endpoint ELISA titres for each animal at the time of tick application. Values represent the last serum dilution where the OD of test sera was ≥ 2 OD of negative control bovine serum donor. Table S2. Tick biological data measurements for normal Rhipicephalus appendiculatus Muguga normal colony ticks from individual animals after multivalent anti-tick vaccination. Table S3. Tick biological data measurements for Rhipicephalus appendiculatus Muguga ‘low-line’ ticks from individual animals after multivalent anti-tick vaccination. Table S4. Summary of the East Coast fever symptoms in individual animals after exposure to 30 ‘low line’ Muguga ticks infected with Theileria parva. (DOCX 43 kb) [file 13071_2016_1774_MOESM1_ESM.docx]

**Additional file 1: Table S1.** Endpoint ELISA titres for each animal at the time of tick application. Values represent the last serum dilution where the OD of test sera was ≥ 2 OD of negative control bovine serum donor

| **Animal number** | **Pool 1** | | | |  | **Pool 2** | | |  |
| --- | --- | --- | --- | --- | --- | --- | --- | --- | --- |
|  | **p67** | **TRP 18-89** | **Subolesin** | **Histamine binding protein F2** |  | **Histamine binding protein M** | **TRP full length** | **Histamine binding protein F1** |  |
| Vaccinated (*n* = 20) | | | | | | | | | |
| BF002 | 12,000 | 64,000 | 12,000 | 64,000 |  | 64,000 | 16,000 | 64,000 |  |
| BF003 | 12,000 | 64,000 | 32,000 | 32,000 |  | 64,000 | 8,000 | 32,000 |  |
| BF005 | 12,000 | 32,000 | 16,000 | 16,000 |  | 32,000 | 8,000 | 16,000 |  |
| BF006 | 2,000 | 16,000 | 32,000 | 16,000 |  | 32,000 | 16,000 | 16,000 |  |
| BF012 | 8,000 | 16,000 | 32,000 | 16,000 |  | 32,000 | 16,000 | 16,000 |  |
| BF016 | 4,000 | 16,000 | 16,000 | 32,000 |  | 8,000 | 4,000 | 32,000 |  |
| BF017 | 12,000 | 64,000 | 64,000 | 32,000 |  | 32,000 | 16,000 | 32,000 |  |
| BF021 | 12,000 | 32,000 | 64,000 | 32,000 |  | 64,000 | 16,000 | 32,000 |  |
| BF022 | 12,000 | 16,000 | 16,000 | 16,000 |  | 32,000 | 8,000 | 16,000 |  |
| BF024 | 2,000 | 32,000 | 32,000 | 32,000 |  | 16,000 | 4,000 | 32,000 |  |
| BF025 | 32,000 | 32,000 | 64,000 | 32,000 |  | 32,000 | 16,000 | 32,000 |  |
| BF026 | 4,000 | 32,000 | 32,000 | 32,000 |  | 16,000 | 2,000 | 32,000 |  |
| BF028 | 8,000 | 16,000 | 16,000 | 16,000 |  | 32,000 | 16,000 | 16,000 |  |
| BF029 | 2,000 | 2,000 | 32,000 | 8,000 |  | 32,000 | 4,000 | 32,000 |  |
| BF030 | 16,000 | 32,000 | 64,000 | 32,000 |  | 64,000 | 16,000 | 32,000 |  |
| BF031 | 4,000 | 2,000 | 64,000 | 32,000 |  | 32,000 | 4,000 | 32,000 |  |
| BF032 | 4,000 | 64,000 | 32,000 | 64,000 |  | 32,000 | 8,000 | 64,000 |  |
| BF034 | 4,000 | 32,000 | 64,000 | 32,000 |  | 32,000 | 12,000 | 32,000 |  |
| BF035 | 4,000 | 32,000 | 64,000 | 32,000 |  | 64,000 | 8,000 | 32,000 |  |
| BF037 | 4,000 | 64,000 | 64,000 | 32,000 |  | 64,000 | 16,000 | 64,000 |  |
| Control (*n* = 10) | | | | | | | | | |
| BF008 | 0 | 0 | 500 | 0 |  | 0 | 0 | 0 |  |
| BF009 | 0 | 0 | 0 | 0 |  | 0 | 500 | 0 |  |
| BF010 | 0 | 500 | 1,000 | 0 |  | 0 | 0 | 500 |  |
| BF011 | 500 | 0 | 500 | 500 |  | 0 | 0 | 0 |  |
| BF013 | 500 | 0 | 500 | 500 |  | 500 | 500 | 0 |  |
| BF014 | 500 | 0 | 0 | 0 |  | 0 | 1,000 | 0 |  |
| BF019 | 0 | 0 | 1,000 | 500 |  | 500 | 0 | 0 |  |
| BF023 | 0 | 500 | 0 | 0 |  | 0 | 1,000 | 0 |  |
| BF027 | 500 | 500 | 1,000 | 0 |  | 0 | 0 | 500 |  |
| BF033 | 0 | 0 | 1,000 | 0 |  | 0 | 0 | 0 |  |

**Additional file 1: Table S2.** Tick biological data measurements for normal *Rhipicephalus appendiculatus* Muguga normal colony ticks from individual animals after multivalent anti-tick vaccination

| **Animal Number** | **Weight of 100 nymphs (g)** | **Nymph molting^a^** | **Adult females recovered^b^** | **Average weight of female (mg)** | **Total egg weight (g)** | **Average egg weight (mg)** |
| --- | --- | --- | --- | --- | --- | --- |
| Vaccinated group (*n* = 20) | | | | | | |
| BF002 | 1.2 | 97 | 49 | 510 | 12.0 | 245 |
| BF003 | 1.2 | 93 | 49 | 516 | 12.3 | 251 |
| BF005 | 1.2 | 100 | 42 | 488 | 10.1 | 240 |
| BF012 | 1.2 | 99 | 50 | 494 | 14.3 | 286 |
| BF025 | 1.2 | 97 | 47 | 514 | 12.4 | 264 |
| BF026 | 1.2 | 99 | 47 | 480 | 7.8 | 160 |
| BF028 | 1.2 | 97 | 39 | 531 | 14.8 | 379 |
| BF029 | 1.2 | 99 | 50 | 537 | 15.2 | 304 |
| BF034 | 1.2 | 98 | 50 | 510 | 12.5 | 250 |
| BF035 | 1.2 | 94 | 37 | 519 | 14.8 | 400 |
| BF006 | 1.2 | 99 | 50 | 600 | 16.4 | 328 |
| BF016 | 1.2 | 98 | 44 | 577 | 16.1 | 370 |
| BF017 | 1.2 | 99 | 50 | 534 | 18.9 | 378 |
| BF021 | 1.2 | 100 | 51 | 384 | 11.6 | 227 |
| BF022 | 1.2 | 99 | 47 | 479 | 10.0 | 444 |
| BF024 | 1.2 | 100 | 50 | 542 | 13.5 | 270 |
| BF030 | 1.2 | 99 | 49 | 597 | 12.7 | 259 |
| BF031 | 1.2 | 96 | 50 | 548 | 12.7 | 254 |
| BF032 | 1.2 | 100 | 50 | 627 | 16.2 | 324 |
| BF037 | 1.2 | 99 | 29 | 569 | 13.1 | 452 |
| Control group (*n* = 10) | | | | | | |
| BF008 | 1.2 | 97 | 47 | 551 | 13.2 | 281 |
| BF009 | 1.2 | 98 | 50 | 494 | 13.1 | 256 |
| BF013 | 1.2 | 100 | 40 | 515 | 7.4 | 185 |
| BF014 | 1.2 | 97 | 48 | 570 | 16.3 | 340 |
| BF023 | 1.2 | 97 | 45 | 544 | 7.6 | 170 |
| BF010 | 1.2 | 98 | 50 | 525 | 12.6 | 242 |
| BF011 | 1.2 | 96 | 46 | 541 | 12.5 | 272 |
| BF019 | 1.2 | 99 | 44 | 582 | 11.3 | 257 |
| BF027 | 1.2 | 99 | 44 | 584 | 10.3 | 234 |
| BF033 | 1.2 | 98 | 45 | 571 | 10.2 | 227 |

^a^Number successfully molting of a random population of 100 ticks monitored

^b^Number of replete female ticks recovered of 50 applied

**Additional file 1: Table S3.** Tick biological data measurements for *Rhipicephalus appendiculatus* Muguga ‘low-line’ ticks from individual animals after multivalent anti-tick vaccination

| **Animal Number** | **Number of replete adult females of 30 applied** | **Total weight of replete females (g)** | **Total egg weight (g)** | **Average egg weight per female (mg)** |
| --- | --- | --- | --- | --- |
| Vaccinated group (*n* = 19*) | | |  |  |
| BF002 | 12 | 1.8 | 0.8 | 67 |
| BF003 | 7 | 1.0 | 0.7 | 100 |
| BF005 | 23 | 4.7 | 0.7 | 30 |
| BF012 | 18 | 5.1 | 0.5 | 28 |
| BF025 | 25 | 6.0 | 2.0 | 80 |
| BF026 | 6 | 1.0 | 0.5 | 83 |
| BF028 | 15 | 4.2 | 0.8 | 53 |
| BF029 | 15 | 4.7 | 0.8 | 53 |
| BF034 | 27 | 6.9 | 1.2 | 44 |
| BF035 | 8 | 2.2 | 0.7 | 88 |
| BF006 | 13 | 3.3 | 0.6 | 46 |
| BF016 | 7 | 1.6 | 0.7 | 100 |
| BF017 | 8 | 2.8 | 1.5 | 188 |
| BF021 | 14 | 3.8 | 1.0 | 71 |
| BF022 | 19 | 4.5 | 2.0 | 105 |
| BF024 | 20 | 5.5 | 1.4 | 70 |
| BF030 | 27 | 7.1 | 1.8 | 67 |
| BF031 | 21 | 6.1 | 1.0 | 48 |
| BF032 | 13 | 3.9 | 0.3 | 23 |
| Control group (*n* = 10) | |  |  |  |
| BF008 | 32 | 11.0 | 3.2 | 100 |
| BF009 | 10 | 2.7 | 0.6 | 60 |
| BF013 | 6 | 1.6 | 0.9 | 150 |
| BF014 | 12 | 3.0 | 0.8 | 67 |
| BF023 | 24 | 7.1 | 1.4 | 58 |
| BF010 | 17 | 4.3 | 1.1 | 65 |
| BF011 | 21 | 5.0 | 1.5 | 71 |
| BF019 | 20 | 5.8 | 1.7 | 85 |
| BF027 | 22 | 6.0 | 1.0 | 45 |
| BF033 | 14 | 3.7 | 1.8 | 129 |

*Animal number 35 died of severe East Coast fever before tick feeding was complete and is not included in the infected tick analysis

**Additional file 1: Table S4.** Summary of the East Coast fever symptoms in individual animals after exposure to 30 ‘low line’ Muguga ticks infected with *Theileria parva*

| **Animal ID** | **Pyrexia^a,b^** | **Regional lymph node parasitosis^a^** | **Contra-lateral lymph node parasitosis^a^** | **Piroplasm^a^** | **p104 PCR** | **Positive PIM ELISA reading^c^** |
| --- | --- | --- | --- | --- | --- | --- |
|  |  |  |  |  |  |  |
|  |  |  |  |  |  |  |
| **Vaccinated** | ***n* = 15** | ***n* = 13** | ***n* = 8** | ***n* = 5** |  |  |
| BF002 | 10 (2) |  |  |  | POS | 37 |
| BF003 |  |  |  |  | NEG | 39 |
| BF005 | 17 (3) | 14 (4) |  |  | POS | 34 |
| BF006 | 5 (12) | 10 (1) |  |  | POS | 58 |
| BF012 | 6 (9) |  |  |  | POS | 42 |
| BF016 | 9 (8) |  |  |  | POS | 56 |
| BF017 | 10 (8) | 14 (7) | 16 (5) | 17 (4) | POS | 58 |
| BF021 | 9 (11) | 10 (9) | 15 (6) | 17 (4) | POS | 88 |
| BF025 |  | 16 (2) | 18 (1) |  | POS | 27 |
| BF026 | 14 (1) | 15 (3) |  |  | POS | 55 |
| BF028 | 3 (2) | 16 (2) |  |  | POS | 25 |
| BF029 | 16(5) | 15 (4) | 18 (1) |  | POS | 42 |
| BF030 |  |  |  |  | POS | 43 |
| BF031 | 15 (5) | 15 (3) | 16 (1) |  | POS | 30 |
| BF032 | 7 (9) | 10 (4) |  | 20 (1) | POS | 21 |
| BF034 | 17 (1) | 14 (6) | 16 (4) | 17 (2) | POS | 40 |
| BF035 | 10 (4) | 13 (2) | 14 (1) |  | POS | 55 |
| BF037 | 6 (7) | 14 (7) | 16 (5) | 17 (4) | POS | 55 |
| **Control** | ***n* = 9** | ***n* = 9** | ***n* = 6** | ***n* = 4** |  |  |
| BF008 | 15 (6) | 15 (5) | 18 (2) |  | POS | 77 |
| BF009 | 17 (4) | 15 (3) | 18 (1) |  | POS | 57 |
| BF010 | 9 (10) | 12 (6) | 16 (4) | 17 (4) | POS | 78 |
| BF011 | 16 (6) | 10 (9) | 16 (5) | 17 (4) | POS | 90 |
| BF013 | 2(1) | 15 (3) | 19 (1) |  | POS | 49 |
| BF014 | 14 (3) | 20 (1) |  | 16 (1) | POS | 59 |
| BF019 | 7 (5) |  |  |  | NEG | 39 |
| BF023 |  | 16 (2) |  |  | POS | 28 |
| BF027 | 5(14) | 13 (2) | 16 (2) | 18 (3) | POS | 27 |
| BF033 | 15 (1) | 10 (2) |  |  | POS | 30 |

^a^Values are displayed as the day of first detection (duration observed in days). Where symptoms were not observed in an animal, no values are reflected

^b^Pyrexia was defined as rectal temperature exceeding 39.5 °C

^c^The average Percentage Positive (PP) value calculated as the (OD of test sample/OD of strong positive) ×100
